# Commentary: Bradykinin-Mediated Angioedema: An Update of the Genetic Causes and the Impact of Genomics

**DOI:** 10.3389/fgene.2020.00304

**Published:** 2020-04-03

**Authors:** Roger Colobran

**Affiliations:** ^1^Immunology Division, Department of Cell Biology, Physiology and Immunology, Hospital Universitari Vall d'Hebron, Vall d'Hebron Research Institute, Autonomous University of Barcelona, Barcelona, Spain; ^2^Department of Clinical and Molecular Genetics, Hospital Universitari Vall d'Hebron, Barcelona, Spain; ^3^Jeffrey Model Foundation Excellence Center, Barcelona, Spain

**Keywords:** hereditary angioedema, plasminogen, mutation, HGVS nomenclature, genetic variant

Hereditary angioedema (HAE) is a rare autosomal-dominant genetic disease characterized by recurrent episodes of edema of the deep dermis and mucosal/submucosal tissues, and less frequently, potentially life-threatening laryngeal edema. The estimated worldwide prevalence of HAE is 1:50,000, but this figure may be even higher, as patients with this condition are often misdiagnosed or undiagnosed (Bernstein, 2018). The genetic bases of HAE were initially associated with mutations in *SERPING1*, the gene that encodes the C1-inhibitor (C1-INH) protein. In the absence of adequate functional C1-INH, the plasma contact system is insufficiently regulated, with consequent generation of bradykinin, the primary mediator of swelling in HAE. Now, more than 20 years since this genetic cause was identified (Bissler et al., 1997), the latest update includes 748 different *SERPING1* variants associated with HAE (Ponard et al., 2019). Nonetheless, in addition *SERPING1*, which still accounts for the majority of genetic defects underlying HAE, an increasing number of new causal genes have been identified, especially in the last few years. The genes that cause HAE in patients with normal C1-INH levels are coagulation factor 12 gene (*F12*) (discovered in 2006) (Dewald and Bork, 2006), angiopoietin-1 (*ANGPT1*) and plasminogen gene (*PLG*) (discovered in 2018) (Bafunno et al., 2018; Bork et al., 2018b), and kininogen-1 gene (*KNG1*) (discovered in 2019) (Bork et al., 2019).

I read with great interest the article by Marcelino-Rodriguez et al. on the genetic causes of bradykinin-mediated angioedema (Marcelino-Rodriguez et al., 2019). These authors comprehensively reviewed all HAE causal genes, including those most recently described, and emphasized the crucial role of next generation sequencing (NGS), specifically whole-exome sequencing (WES), in these recent discoveries. When they dealt with *PLG*, one of the recently described HAE causal genes, they focused on the p.Lys330Glu mutation, a recurrent mutation described in more than 20 independent families from different European countries and Japan (Belbézier et al., 2018; Bork et al., 2018b; Germenis et al., 2018; Yakushiji et al., 2018; Recke et al., 2019), and also mentioned the p.Lys311Glu variant, described in three patients by Dewald (2018). I would like to clarify that Lys330Glu and Lys311Glu are actually two different names for the same genetic variant. Lys330Glu refers to amino acid residue 330 of the primary translated protein, and Lys311Glu to amino acid residue 311 of the mature protein. The main reason for this discrepancy is the 19-amino-acid signal peptide present in the PLG protein, which allows it to be secreted to the extracellular region (Figure 1). The HGVS (Human Genome Variation Society) recommendations for describing sequence variants state the following: “*A protein reference sequence should represent the primary translation product, not a processed mature protein, and thus includes the starting methionine, any signal peptide sequences, etc*.” (den Dunnen et al., 2016). Therefore, the correct name for this *PLG* mutation at the protein level is p.Lys330Glu.

**Figure 1:**
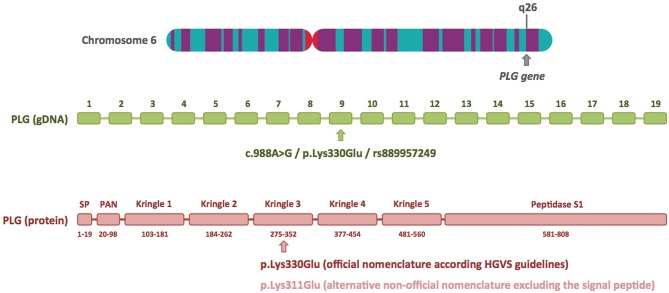
Plasminogen gene structure and location of the c.988A>G/p.Lys330Glu mutation at the DNA and protein level. The plasminogen gene (*PLG*) has 19 exons and is located on the long arm of chromosome 6 (6q26). The HAE-associated c.988A>G/p.Lys330Glu mutation is located in exon 9 and is included in the dbSNP database (ID rs889957249). The diagram of the plasminogen protein shows its different domains and numbering of the amino acids (based on UniProtKB: P00747). The p.Lys330Glu mutation is located in the kringle 3 domain. The alternative nomenclature, p.Lys311Glu, is based on the mature protein, and it excludes the first 19 amino acids of the signal peptide (SP). The chromosome 6 image was obtained from the Human Genome Project Archive.

At the cDNA level, different nomenclature was used by Bork et al. in the article that first identified this mutation (c.988A>G) and by Georg Dewald (c.1100A>G) (Bork et al., 2018b; Dewald, 2018). Nucleotide positions 988 and 1100 refer to the same position in the sequence of the canonical plasminogen transcript (NBCI reference sequence NM_000301.3): 988 is the position relative to the start codon, whereas 1100 is the position relative to the transcription start site, containing 112 untranslated nucleotides before the first methionine. Again, the HGVS recommendations state: “*cDNA numbering starts with ‘c.1' at the A of the ATG translation initiation (start) codon*”. Therefore, the correct name for the *PLG* mutation at the cDNA level is c.988A>G. Although Georg Dewald repeatedly uses the alternative nomenclature to describe the PLG mutation in his report (c.1100A>G/p.Lys311Glu), it must be said that at one point he indicates that this variant can be also named c.988A>G/p.Lys330Glu (Dewald, 2018). However, this brief comment may go unnoticed by readers, and this may have occurred in the case of Marcelino-Rodriguez et al. (2019).

Another confusing factor in reporting the *PLG* defect in HAE is that in the original article by Bork at al. describing the p.Lys330Glu mutation, the name of the mutation at the cDNA level was incorrect in the abstract and results section. Instead of c.988A>G, it appeared as c.9886A>G several times in the text (likely an involuntary typographic error). The authors noted the error and published a corrigendum several months later (Bork et al., 2018a).

The aim of this commentary is simply to clarify that, to date, only one *PLG* mutation causing HAE-PLG has been discovered (c.988A>G/p.Lys330Glu), although the aforementioned nomenclature-related factors may give a different impression. Haplotype analysis demonstrated a founder effect that likely explains recurrence of this mutation in more than 20 independent families identified so far (Bork et al., 2018b). The existence of symptom-free carriers indicates that clinical penetrance is incomplete. Although the pathomechanism of HAE-PLG has not been elucidated as yet, there is evidence pointing to bradykinin as the main mediator of edema in this condition; that is, bradykinin-2 receptor antagonist has been effective in shortening acute attacks in several patients (Belbézier et al., 2018; Bork et al., 2018b; Recke et al., 2019). Whether or not other variants in *PLG* are a cause of HAE remains to be determined. In any case, in the current era of NGS capability, systematic use of the HGVS nomenclature is essential when reporting genetic variants to avoid misunderstandings on dissemination of the information.

Finally, another inaccuracy regarding the article by Marcelino-Rodriguez et al. can be pointed out. In Table 1, they selected 12 articles described as “*genetic studies of acquired bradykinin-mediated angioedema*.” However, the first manuscript from this list is, as its title claims (*Hereditary Angioedema with Normal C1 Inhibitor and F12 Mutations in 42 Brazilian Families*), a study of HAE patients with normal C1-INH and mutations in *F12* gene (Veronez et al., 2018), and not a study of acquired angioedema.

## Author Contributions

The author confirms being the sole contributor of this work and has approved it for publication.

### Conflict of Interest

The author declares that the research was conducted in the absence of any commercial or financial relationships that could be construed as a potential conflict of interest.
